# Supplementary material for: Trends in HIV incidence between 2013–2019 and association of baseline factors with subsequent incident HIV among gay, bisexual, and other men who have sex with men attending sexual health clinics in England: A prospective cohort study
Source: PLoS Med. 2021 Jun 18;18(6):e1003677. doi: 10.1371/journal.pmed.1003677 (PMC8253400; doi:10.1371/journal.pmed.1003677)
Supplement: S1 Checklist — (DOC) [file pmed.1003677.s001.doc]

STROBE Statement—Checklist of items that should be included in reports of ***cohort studies***

|  | Item No | Recommendation | Response |
| --- | --- | --- | --- |
| **Title and abstract** | 1 | (*a*) Indicate the study’s design with a commonly used term in the title or the abstract | **Title:** “a prospective cohort study” |
|  |  | (*b*) Provide in the abstract an informative and balanced summary of what was done and what was found | **Section:** Abstract  **Subsections:** Methods and findings, Conclusions |
| Introduction | | |  |
| Background/rationale | 2 | Explain the scientific background and rationale for the investigation being reported | **Section:** Introduction  **Paragraphs:** 1 |
| Objectives | 3 | State specific objectives, including any prespecified hypotheses | **Section:** Introduction  **Paragraphs:** 2 |
| Methods | | |  |
| Study design | 4 | Present key elements of study design early in the paper | **Section:** Methods  **Subsection:** Study design and participants |
| Setting | 5 | Describe the setting, locations, and relevant dates, including periods of recruitment, exposure, follow-up, and data collection | **Section:** Methods  **Subsection:** Study design and participants |
| Participants | 6 | (*a*) Give the eligibility criteria, and the sources and methods of selection of participants. Describe methods of follow-up | **Section:** Methods  **Subsection:** Study design and participants |
|  |  | (*b*) For matched studies, give matching criteria and number of exposed and unexposed | N/A |
| Variables | 7 | Clearly define all outcomes, exposures, predictors, potential confounders, and effect modifiers. Give diagnostic criteria, if applicable | **Section:** Methods  **Subsections:** Baseline measures, Time-updated measures, Ascertainment of incident HIV |
| Data sources/ measurement | 8* | For each variable of interest, give sources of data and details of methods of assessment (measurement). Describe comparability of assessment methods if there is more than one group | **Section:** Methods  **Subsections:** Study design and participants, Ascertainment of incident HIV |
| Bias | 9 | Describe any efforts to address potential sources of bias | **Section:** Methods  **Subsection:** Ascertainment of incident HIV |
| Study size | 10 | Explain how the study size was arrived at | Sample size has been reported in the previous paper (*Sewell et al, 2016*) |
| Quantitative variables | 11 | Explain how quantitative variables were handled in the analyses. If applicable, describe which groupings were chosen and why | **Section:** Methods  **Subsection:** Statistical analysis |
| Statistical methods | 12 | (*a*) Describe all statistical methods, including those used to control for confounding | **Section:** Methods  **Subsection:** Statistical analysis |
|  |  | (*b*) Describe any methods used to examine subgroups and interactions | **Section:** Methods  **Subsection:** Statistical analysis |
|  |  | (*c*) Explain how missing data were addressed | **Section:** Methods  **Subsection:** Statistical analysis |
|  |  | (*d*) If applicable, explain how loss to follow-up was addressed | **Section:** Methods  **Subsection:** Statistical analysis |
|  |  | (*e*) Describe any sensitivity analyses | **Section:** Methods  **Subsection:** Statistical analysis |
| Results | | |  |
| Participants | 13* | (a) Report numbers of individuals at each stage of study—eg numbers potentially eligible, examined for eligibility, confirmed eligible, included in the study, completing follow-up, and analysed | **Section:** Results  **Subsection:** Characteristics of the participants |
|  |  | (b) Give reasons for non-participation at each stage | Details on non-participation has been reported in the previous paper (*Sewell et al, 2016*) |
|  |  | (c) Consider use of a flow diagram | flow diagram has been reported in the previous paper (*Sewell et al, 2016*) |
| Descriptive data | 14* | (a) Give characteristics of study participants (eg demographic, clinical, social) and information on exposures and potential confounders | **Section:** Results  **Subsection:** Characteristics of the participants |
|  |  | (b) Indicate number of participants with missing data for each variable of interest | **Section:** Results  **Subsection:** Characteristics of the participants, Table 1 |
|  |  | (c) Summarise follow-up time (eg, average and total amount) | **Section:** Results  **Subsection:** Characteristics of the participants, Trends in HIV incidence, Table 1, Table 2 |
| Outcome data | 15* | Report numbers of outcome events or summary measures over time | **Section:** Results  **Subsection:** Trends in HIV incidence, Table 1, Table 2 |
| Main results | 16 | (*a*) Give unadjusted estimates and, if applicable, confounder-adjusted estimates and their precision (eg, 95% confidence interval). Make clear which confounders were adjusted for and why they were included | **Section:** Results  **Subsection:** Association of baseline factors with incident HIV, HIV incidence and longitudinal associated factors among men in the AURAH2 cohort who completed at least one online follow-up questionnaire of the participants, Table 1, Table 3, Supplementary table 2 |
|  |  | (*b*) Report category boundaries when continuous variables were categorized | **Section:** Results  **Subsection:** Association of baseline factors with incident HIV, HIV incidence and longitudinal associated factors among men in the AURAH2 cohort who completed at least one online follow-up questionnaire of the participants, Table 1, Table 3, Supplementary table 2 |
|  |  | (*c*) If relevant, consider translating estimates of relative risk into absolute risk for a meaningful time period | N/A |
| Other analyses | 17 | Report other analyses done—eg analyses of subgroups and interactions, and sensitivity analyses | **Section:** Results  **Subsection:** HIV incidence and longitudinal associated factors among men in the AURAH2 cohort who completed at least one online follow-up questionnaire of the participants, Table 3, Supplementary table 2 |
| Discussion | | |  |
| Key results | 18 | Summarise key results with reference to study objectives | **Section:** Discussion  **Paragraph:** 1 |
| Limitations | 19 | Discuss limitations of the study, taking into account sources of potential bias or imprecision. Discuss both direction and magnitude of any potential bias | **Section:** Discussion  **Paragraph:** 9 |
| Interpretation | 20 | Give a cautious overall interpretation of results considering objectives, limitations, multiplicity of analyses, results from similar studies, and other relevant evidence | **Section:** Discussion  **Paragraph:** 9 |
| Generalisability | 21 | Discuss the generalisability (external validity) of the study results | **Section:** Discussion  **Paragraph:** 9 |
| Other information | | |  |
| Funding | 22 | Give the source of funding and the role of the funders for the present study and, if applicable, for the original study on which the present article is based | **Section:** Funding |

*Give information separately for exposed and unexposed groups.

**Note:** An Explanation and Elaboration article discusses each checklist item and gives methodological background and published examples of transparent reporting. The STROBE checklist is best used in conjunction with this article (freely available on the Web sites of PLoS Medicine at http://www.plosmedicine.org/, Annals of Internal Medicine at http://www.annals.org/, and Epidemiology at http://www.epidem.com/). Information on the STROBE Initiative is available at http://www.strobe-statement.org.
