# Supplementary material for: Trends in HIV incidence between 2013–2019 and association of baseline factors with subsequent incident HIV among gay, bisexual, and other men who have sex with men attending sexual health clinics in England: A prospective cohort study
Source: PLoS Med. 2021 Jun 18;18(6):e1003677. doi: 10.1371/journal.pmed.1003677 (PMC8253400; doi:10.1371/journal.pmed.1003677)
Supplement: S1 Analyses Plan — (PDF) [file pmed.1003677.s002.pdf]

Longitudinal analysis of new HIV infections and their predictors among MSM in England 2013 – 2019: the AURAH2 study

Data Analysis Plan

This data analysis plan summarizes the strategies and general statistical considerations that will be employed to address specific objectives and aims on the assessment of the trends in HIV incidence among MSM in the AURAH2 study.

1. Aim

The aim of the following guide is to set forth a statistical analysis plan to answer the following questions:

- What has been the trend in HIV incidence among MSM in the AURAH2 study?
- Which factors predict HIV new infections?
- What is the incidence among MSM who reported having recent condom-less anal sex (CLS) and other high-risk sexual behaviours?
- Is there any evidence that the use of PrEP explains any decline in HIV incidence?

2. Methods

This was a prospective longitudinal study of HIV negative MSM recruited from Genito-Urinary Medicine (GUM) clinics in England. To verify the numbers of HIV diagnoses reported by the participants and to discover any diagnoses within cohort that have not been reported, a linking process between the AURAH2 data and national HIV surveillance data managed by Public Health England (PHE) has been carried out to all 1162 participants who completed the baseline paper questionnaire. The matching process took the form of a deterministic, hierarchical algorithm using the Stata software.

Study Measures

Outcome variable: HIV diagnosis

Participants' self-reported HIV status, HIV testing history, and date of first positive HIV test seroconversion will be extracted from online follow-up questionnaires. Date of HIV diagnosis will be extracted and matched to final dataset from Public Health England (PHE).

Main exposure: sexual behaviours and PrEP use variables

Participants' information on CLS activities and other sexual behaviours will be extracted from baseline, four monthly, and annual questionnaires, while information on PrEP use will be extracted from baseline and annual questionnaires only.

Other covariates: demographics and lifestyle variables

Participants' socio-demographic and lifestyle characteristics data are available from baseline and follow-up questionnaires and include assessment of age, ethnicity, migrancy, education, employment, sexuality, relationship status, recreational drug use and chemsex, mental health, STI diagnoses, and alcohol consumption.

Treatment of missing data

Two methods will be used for dealing with missing data, which are:

- Listwise deletion (complete case analysis): analyses will be restricted to individuals with observed data, for age and socio-demographic and partnership status characteristics It's a reasonable solution when cases with missing data are implicitly assumed to be no different than the complete cases, that they are a purely random subset of the data (missing completely at random).
- Treating missing responses as 'No' for sexual behaviour and lifestyle characteristics.

Sequence of planned analyses

(Recommended statistics for all analysis, as applicable: the indication of data normality, count (n), mean, standard deviation, geometric mean (95%CI), median (i.e. p50)).

- Sample characteristics
  Descriptive statistics of the study population included in the analyses will be done using baseline data.

- Trends in HIV incidence
  - The HIV incidence rates will be defined as the number of HIV diagnosis during follow-up divided by the total number of person-years (PYs) of observation.
  - The person-year contribution will be calculated for participants who seroconverted (time between the baseline and the date of HIV diagnosis), for those who remained negative (time between the baseline visit and the censoring date; the date of linking data with PHE completion), and for those who died (time between the baseline visit and the date of death).
  - Cox proportional hazard with diagnosis as the binary outcome, and calendar year as a continuous variable will be modelled to assess the incidence trends.

- Incidence rates per calendar year
  - A new dataset consists of each participants' person years that are divided up into calendar time periods and seroconversion events in each period of person years' count will be created.
  - Visualisation: differences in rates will be shown through a figure, with period as the parameter of interest (the plot of rates with 95% CI will be made per calendar year), and a table that shows the incidence rates in each calendar year.

- Incidence among MSM who reported PrEP use and or high risk sexual behaviours
  - Incidence rates will be stratified by sexual behaviour, other health and lifestyle, and socio-demographic characteristics.
  - Prevalence of PrEP use and other high risk sexual behaviours will be calculated to determine the calendar-year trends; trends will be assessed using logistic regression fitted with Generalized Estimating Equation (GEE), to take into account repeated measurements within individuals.
  - Results from both Cox proportional hazard and Poisson regression will also be used to answer whether there is any evidence that the use of PrEP explains any decline in HIV incidence.

- Visualisation: differences in incidence rates will be shown in a table, and the prevalence (trends) of PrEP use and sexual behaviours will be shown in graphs / plots and or tables

- Factors that predict new infections
  - Data will initially be explored through univariate descriptive statistics (frequencies for categorical variables, central tendency, dispersion, estimates´ errors).
  - Next, to create more complex models with the inclusion of many time-updated variables that were collected in a longitudinal design, unadjusted and adjusted Poisson regression models will be used.
  - Unadjusted and adjusted Cox proportional hazard models will be employed to assess association of baseline characteristics and HIV incidence

Analysis software

All analyses will be done using STATA SE version 15.1 (StataCorp LLC, Texas)
