# Supplementary material for: Trends in HIV incidence between 2013–2019 and association of baseline factors with subsequent incident HIV among gay, bisexual, and other men who have sex with men attending sexual health clinics in England: A prospective cohort study
Source: PLoS Med. 2021 Jun 18;18(6):e1003677. doi: 10.1371/journal.pmed.1003677 (PMC8253400; doi:10.1371/journal.pmed.1003677)
Supplement: S1 Table — (DOCX) [file pmed.1003677.s003.docx]

**S1 Table. Baseline socio-demographic, health and lifestyle characteristics, sexual behaviour, and PrEP and PEP use among participants who completed the baseline, four-monthly, and annual questionnaire in the AURAH2 study, 2013 – 2018***

|  | Completing baseline questionnaire  (all paper, at clinic of recruitment, N=1162) | | Completing at least one follow-up questionnaire  (all online, N=622) | | Completing at least one annual questionnaire  (all online, N=483) | |
| --- | --- | --- | --- | --- | --- | --- |
|  | **N** | **n (%)** | **N** | **n (%)** | **N** | **n (%)** |
| Age category  <25  25 – 29  30 – 34  35 – 39  40 – 44  ≥45  Mean age (SD)  Median age  p<0.001** | 1153 | 275 (23·9%)  207 (17·9%)  227 (19·6%)  156 (13·6%)  121 (10·5%)  167 (14·5%)  **34 years (10·4)**  **31 years** | 613 | 132 (21·5%)  86 (14·0%)  121 (19·8%)  89 (14·5%)  70 (11·4%)  114 (18·6%)  **34 years (11·3)**  **33 years** | 479 | 103 (21·5%)  69 (14·4%)  87 (18·2%)  68 (14·2%)  55 (11·5%)  97 (20·2%)  **35 years (11·2)**  **33 years** |
| Born in the UK & ethnicity  Yes, White  Yes, Other ethnicity^§^  No, White  No, Other ethnicity  p=0.204** | 1150 | 568 (49·4%)  60 (5·2%)  374 (32·5%)  148 (12·9%) | 611 | 317 (51·9%)  29 (4·7%)  195 (31·9%)  70 (11·5%) | 479 | 262 (54·7%)  20 (4·2%)  149 (31·1%)  48 (10·0%) |
| Sexual identity  Gay  Bisexual / other  p=0.179** | 1150 | 1076 (93·6%)  74 (6·4%) | 614 | 581 (94·6%)  33 (5·4%) | 480 | 455 (94·8%)  25 (5·2%) |
| University education  Yes  No  p=0.017** | 1146 | 853 (74·4%)  293 (25·6%) | 607 | 462 (76·4%)  145 (23·8%) | 475 | 359 (75·6%)  116 (24·4%) |
| Employed  Yes  No  p=0.01** | 1149 | 952 (82·9%)  197 (17·1%) | 607 | 508 (83·7%)  99 (16·3%) | 475 | 395 (83·2%)  80 (16·8%) |
| Money to cover basic needs  All of the time  Most of the time  Sometimes / No  p<0.001** | 1158 | 896 (77·4%)  194 (16·8%)  68 (5·8%) | 618 | 509 (82·4%)  81 (13·1%)  28 (4·5%) | 481 | 404 (84·0%)  58 (12·1%)  19 (3·9%) |
| Housing status^₴^  Home owner  Renting  Unstable / other  p<0.001** | 1147 | 314 (27·4%)  680 (59·3%)  153 (13·3%) | 607 | 200 (33·0%)  328 (54·0%)  79 (13·0%) | 475 | 168 (35·4%)  250 (52·6%)  57 (12·0%) |
| Ongoing relationship  Yes  No  p=0.312** | 1159 | 465 (40·2%)  693 (59·8%) | 619 | 257 (41·5%)  362 (58·5%) | 481 | 202 (42·0%)  279 (58·0%) |
| Recent HIV test  No  Yes | 1159 | 322 (27·8%)  837 (72·2%) | 619 | 155 (25·2%)  395 (74·8%) | 481 | 123 (25·6%)  358 (74·4%) |
| CLS in the past 3 months  No  Yes | 1159 | 418 (36·1%)  741 (63·9%) | 619 | 224 (36·2%)  395 (63·8%) | 480 | 172 (35·8%)  308 (64·2%) |
| CLS with more than 2 partners in past 3 months  No  Yes | 1159 | 749 (64·6%)  410 (35·4%) | 619 | 385 (62·2%)  234 (37·8%) | 480 | 299 (62·3%)  181 (37·7%) |
| Group sex in the past 3 months  No  Yes | 1159 | 659 (56·9%)  500 (43·1%) | 619 | 327 (52·8%)  292 (47·2%) | 480 | 247 (51·5%)  233 (48·5%) |
| Recreational drug use  No  Non-injection drug and non-chemsex use  Chemsex-related drug use (no-injection)  Injection drug use | 1159 | 464 (40·0%)  336 (29·0%)  321 (27·7%)  38 (3·3%) | 619 | 229 (37·0%)  179 (28·9%)  192 (31·0%)  19 (3·1%) | 480 | 181 (37·7%)  145 (30·2%)  139 (29·0%)  15 (3·1%) |
| STI diagnoses  No  Yes | 1159 | 719 (62·0%)  440 (38·0%) | 619 | 384 (62·0%)  235 (38·0%) | 480 | 297 (61·8%)  183 (38·1%) |
| Higher risk alcohol consumption (modified WHO AUDIT-C ≥6)  No  Yes | 1159 | 1008 (87·0%)  151 (13·0%) | 619 | 537 (86·8%)  82 (13·2%) | 483 | 418 (86·5%)  65 (13·5%) |
| Depressive symptoms (PHQ-9 score ≥10)  No  Yes | 1159 | 1018 (87·8%)  141 (12·2%) | 619 | 544 (87·9%)  75 (12·1%) | 483 | 424 (87·8%)  59 (12·2%) |
| Anxiety symptoms (GAD& score ≥10)  No  Yes | 1159 | 1033 (89·1%)  126 (10·9%) | 619 | 562 (90·8%)  57 (9·2%) | 483 | 440 (91·1%)  43 (8·9 %) |
| PEP use in the past 12 months  No  Yes | 1159 | 919 (79·3%)  240 (20·7%) | 619 | 496 (80·1%)  123 (19·9%) | 481 | 387 (80·5%)  94 (19·5%) |
| PrEP use in the past 12 months  No  Yes | 1159 | 1101 (95·0%)  58 (5·0%) | 619 | 589 (95·2%)  30 (4·8%) | 481 | 246 (95·6%)  21 (4·4%) |
| * Data from baseline paper questionnaire  ** p-value from χ2 test for differences between men who completed only baseline questionnaire (n= 540) and men who continued on the study by completing at least an online questionnaire (n=622)  *Abbreviations:*  *GBMSM: gay, bisexual, and other men who have sex with men; CLS: condomless anal sex; STI: sexually transmitted infections; PEP: post-exposure prophylaxis; PrEP: pre-exposure prophylaxis; WHO-AUDIT: world health organization – alcohol use disorders identification test; PHQ-9: patient health questionnaire - 9; GAD–7: generalised anxiety disorder-7* | | | | | | |
