# Supplementary material for: Trends in HIV incidence between 2013–2019 and association of baseline factors with subsequent incident HIV among gay, bisexual, and other men who have sex with men attending sexual health clinics in England: A prospective cohort study
Source: PLoS Med. 2021 Jun 18;18(6):e1003677. doi: 10.1371/journal.pmed.1003677 (PMC8253400; doi:10.1371/journal.pmed.1003677)
Supplement: S2 Table — (DOCX) [file pmed.1003677.s004.docx]

**S2 Table. Adjusted associations of baseline characteristics with incident HIV among 1162 GBMSM participating in the AURAH2 prospective study, 2013 – 2019***

| **Baseline characteristics** | **Adjusted Hazard Ratio (95% CI)** | **p-value** |
| --- | --- | --- |
| **Demographic characteristics** | | |
| **Age at baseline category, years**  <25  25-29  30-34  35-39  40-44  ≥45 | 1140 observations  1 (Ref)  0.63 (0.16 – 2.45)  0.55 (0.14 – 2.13)  2.02 (0.72 – 5.63)  1.40 (0.41 – 4.81)  1.22 (0.39 – 3.86) | 0.272 |
| **Country of birth and ethnicity**  Born in the UK, White  Born on the UK, other ethnicity  Non-UK born, White  Non-UK born, other ethnicity | 1140 observations  1 (Ref)  0.95 (0.12 – 7.49)  2.68 (1.22 – 5.86)  0.81 (0.17 – 3.74) | 0.145 |
| **Sexual Identity**  Gay  Bisexual / other | 1140 observations  1 (Ref)  2.18 (0.76 – 6.29) | 0.148 |
| **Socio-economic characteristics and partnership status** | | |
| **University education**  Yes  Other qualification  No qualification | 1140 observations  1 (Ref)  2.10 (0.98 – 4.50)  **4.84 (1.10 – 21.24)** | **0.011** |
| **Employed**  Yes  No | 1138 observations  1 (Ref)  0.16 (0.02 – 1.18) | 0.072 |
| **Money to cover basic needs**  All of the time  Most of the time  Sometimes / No | 1140 observations  1 (Ref)  0.89 (0.33 – 2.36)  0.46 (0.06 – 3.46) | 0.469 |
| **Housing status**  Renting  Home owner  Unstable or other | 1136 observations  1 (Ref)  3.72 (1.52 – 9.09)  1.15 (0.32 – 4.13) | 0.194 |
| **Ongoing relationship**  Yes, living with partner  Yes, not living with partner  No | 1140 observations  1 (Ref)  0.42 (0.12 – 1.53)  0.58 (0.26 – 1.26) | 0.215 |
| **Sexual / HIV-related behaviour characteristics** | | |
| **HIV test in the past 6 months**  No  Yes | 1140 observations  1 (Ref)  1.53 (0.62 – 3.78) | 0.350 |
| **CLS in the past 3 months**  No  Yes | 1140 observations  1 (Ref)  **3.65 (1.27 – 10.51)** | **0.016** |
| **Number of CLS partners in the past 3 months**  No CLS partners  One CLS partner  2 – 4 partners  5 – 10 partners  More than 10 partner | 1140 observations  1 (Ref)  0.99 (0.22 – 4.48)  **3.81 (1.21 – 11.99)**  **9.83 (2.59 – 37.39)**  **13.81 (4.02 – 47.47)** | **<0.001** |
| **CLS with partners known to be HIV positive in the past 3 months^‡^**  No  Yes | 1140 observations  1 (Ref)  **6.60 (3.15 – 13.80)** | **<0.001** |
| **Sexual role CLS in the past three months**  No CLS / didn’t state which partner  Always insertive  Always receptive  sometimes insertive, sometimes receptive | 1140 observations  1 (Ref)  0.93 (0.17 – 5.10)  2.06 (0.45 – 9.36)  **6.33 (2.16 – 18.54)** | **<0.001** |
| **Number of new sexual partners in the past 12 months~**  0 – 10 new partners  11 – 49 new partners  50 – 99 new partners  100 or more new partners | 1140 observations  1 (Ref)  **3.39 (1.47 – 7.83)**  **4.42 (1.34 – 14.48)**  **4.55 (0.98 – 21.23)** | **0.001** |
| **Group sex in the past 3 months**  No  Yes | 1140 observations  1 (Ref)  **8.89 (3.10 – 25.50)** | **<0.001** |
| **Fisting or sex toys use in the past 3 months**  No  Yes | 1140 observations  1 (Ref)  1.63 (0.79 – 3.34) | 0.185 |
| **Sex for drugs or money in the past 3 months**  No  Yes | 1140 observations  1 (Ref)  2.81 (0.94 – 8.34) | 0.063 |
| **PEP use in the past 12 months**  No  Yes | 1140 observations  1 (Ref)  2.07 (0.98 – 4.37) | 0.058 |
| **PrEP use in the past 12 months**  No  Yes | 1140 observations  1 (Ref)  2.00 (0.60 – 6.64) | 0.259 |
| **Bacterial STI diagnoses in the past 12 months**  No  Yes | 1140 observations  1 (Ref)  **4.02 (1.82 – 8.87)** | **0.001** |
| **Health and lifestyle characteristics** | | |
| **Smoking status**  Never smoked  Ex-smoker  Regular smoker | 1138 observations  1 (Ref)  1.18 (0.49 – 2.85)  1.44 (0.60 – 3.46) | 0.409 |
| **Recreational drug use in the past 3 months**  No  Non-injection drug and non-chemsex use  Chemsex-related drug use (no-injection)  Injection drug use | 1140 observations  1 (Ref)  **3.92 (1.04 – 14.86)**  **6.83 (1.92 – 24.29)**  **29.77 (7.38 – 120.02)** | **<0.001** |
| **Higher risk alcohol consumption (modified WHO AUDIT-C score of ≥6)**  No  Yes | 1140 observations  1 (Ref)  0.90 (0.34 – 2.38) | 0.837 |
| **Depressive symptoms (PHQ-9 score ≥10)**  No  Yes | 1140 observations  1 (Ref)  0.98 (0.34 – 2.85) | 0.971 |
| **Anxiety symptoms (GAD7 score ≥10)**  No  Yes | 1140 observations  1 (Ref)  0.53 (0.12 – 2.25) | 0.389 |
| **Year of enrolment**  2013  2014  2015  2016 | 1140 observations  1 (Ref)  0.33 (0.05 – 2.03)  0.43 (0.10 – 1.88)  0.60 (0.12 – 3.06) | 0.913 |
| **^*^ adjusted for age at baseline, country of birth and ethnicity, sexual identity, and university education**  ***Abbreviations:***  *GBMSM: gay, bisexual, and other men who have sex with men; CLS: condomless anal sex; STI: sexually transmitted infections; PEP: post-exposure prophylaxis; PrEP: pre-exposure prophylaxis; WHO-AUDIT: world health organization – alcohol use disorders identification test; PHQ-9: patient health questionnaire - 9; GAD–7: generalised anxiety disorder-7* | | |
