# Supplementary material for: Trends in HIV incidence between 2013–2019 and association of baseline factors with subsequent incident HIV among gay, bisexual, and other men who have sex with men attending sexual health clinics in England: A prospective cohort study
Source: PLoS Med. 2021 Jun 18;18(6):e1003677. doi: 10.1371/journal.pmed.1003677 (PMC8253400; doi:10.1371/journal.pmed.1003677)
Supplement: S3 Table — (DOCX) [file pmed.1003677.s005.docx]

S3 Table. Associations between ethnicity, education, and employment characteristics with sexual behavior measures at baseline among 1162 GBMSM in AURAH2 study*

|  | | **CLS** | **CLS ≥2 partners** | **Group sex** | **Non-injection chemsex** | **Injection drug use** | **Bacterial STI** | **PEP use** | **PrEP use** |
| --- | --- | --- | --- | --- | --- | --- | --- | --- | --- |
|  | N (%) | % | % | % | % | % | % | % | % |
| **Born in the UK and ethnicity**  Yes, white  Yes, other ethnicity  No, white  No, other ethnicity  **University Education**  Yes  Other qualification  No qualification  **Employed**  Yes  No | 568 (49.4%)  60 (5.2%)  374 (32.5%)  148 (12.9%) | 62.2%  65.0%  64.7%  67.6%  *p=0.626* | 32.6%  38.3%  35.3%  44.6%  *p=0.053* | 42.9%  40.%  44.1%  45.3%  *p=0.893* | 26.8%  20.%  30.2%  29.1%  *p=0.683* | 3.2%  5.0%  3.2%  3.4%  *p=0.683* | 32.4%  42.3%  **44.9%**  39.9%  ***p=0.001*** | 17.6%  25.0%  23.3%  25.7%  *0.054* | 4.9%  6.7%  5.1%  4.7%  *0.945* |
|  | 853 (73.4%)  272 (23.4%)  21 (1.8%) | 62.4%  67.3%  85.7%  ***p=0.038*** | 34.2%  38.6%  38.6%  *p=0.327* | 43.4%  43.8%  33.3%  *p=0.646* | 26.1%  33.1%  28.6%  *p=0.308* | 3.2%  3.7%  0%  *p=0.308* | 36.2%  43.0%  38.1%  *p=0.133* | 20.2%  22.4%  33.3%  *p=0.270* | 4.7%  5.9%  9.5%  *p=0.473* |
|  | 952 (82.9%)  197 (17.1%) | 64.1%  62.9%  *p=0.763* | 36.5%  30.5%  *p=0.109* | 43.9%  41.6%  *p=0.556* | 29.4%  20.3%  ***p=0.009*** | 3.0%  4.6%  *p=0.009* | 38.1%  38.1%  *p=0.988* | 21.1%  19.3%  *p=0.566* | 5.5%  3.1%  *p=0.158* |

*****Pearson χ^2^ test

***Abbreviations:***

*GBMSM: gay, bisexual, and other men who have sex with men; CLS: condomless anal sex; STI: sexually transmitted infections; PEP: post-exposure prophylaxis; PrEP: pre-exposure prophylaxis.*
