# Supplementary material for: Trends in HIV incidence between 2013–2019 and association of baseline factors with subsequent incident HIV among gay, bisexual, and other men who have sex with men attending sexual health clinics in England: A prospective cohort study
Source: PLoS Med. 2021 Jun 18;18(6):e1003677. doi: 10.1371/journal.pmed.1003677 (PMC8253400; doi:10.1371/journal.pmed.1003677)
Supplement: Attachment — Submitted filename: Responses_AURAH2_HIV Incidence 2013-2019_PlosMed_R1.docx [file pmed.1003677.s006.docx]

**Manuscript PMEDICINE-D-21-01009R1**

**Response to the editorial team and reviewers**

**Requests from the editors:**

1. Please revise your title according to PLOS Medicine's style. Your title must be nondeclarative and not a question. It should begin with main concept if possible. Please place the study design ("A prospective cohort study,") in the subtitle (i.e., after a colon).

**Response:** We have revised our manuscript title according to Plos Medicine style (p 1–2).

2. The Data Availability Statement (DAS) requires revision. For each data source used in your study: If the data are not freely available, please describe briefly the ethical, legal, or contractual restriction that prevents you from sharing it. Please also include an appropriate contact (web or email address) for inquiries (again, this cannot be a study author/ co-author).

**Response:** We have revised our Data Availability Statement (p 22).

3. Abstract summary - At this stage, we ask that you reformat your non-technical Author Summary. The Author Summary should immediately follow the Abstract in your revised manuscript. This text is subject to editorial change and should be distinct from the scientific abstract. The summary should be accessible to a wide audience that includes both scientists and non-scientists. Please see our author guidelines for more information.

**Response:** We have now included an author summary section in the revised manuscript (p 4).

4. Abstract:
a) Please structure your abstract using the PLOS Medicine headings (Background, Methods and Findings, Conclusions).

**Response:** We have structured our abstract according to Plos Medicine style (p 2–3), all numbers presented in the abstract are present and identical to numbers presented in the main manuscript text, and we do not exceed 500 words.

b) Please include the actual amounts or percentages of relevant outcomes, not just hazard ratios or relative risks.

**Response**: The actual amounts of relevant outcomes have been included in the abstract.

c) Please include the important dependent variables that are adjusted for in the analyses.

**Response:** The variables have been included in the Methods and findings (p2, lines 46 – 47).

d) Please quantify the main results (with p values in addition to 95% CI).

**Response:** We have included p-values in addition to 95% CIs to the abstract.

e) Please include a summary of adverse events if these were assessed in the study.

**Response:** We were not studying an intervention – HIV itself is an adverse event.

f) In the last sentence of the Abstract Methods and Findings section, please describe the main limitation(s) of the study's methodology.

**Response:** We have added a brief sentence of the main limitations of the AURAH2’s methodology in the Methods and Findings section (p3, lines 69 – 70).

g) Abstract Conclusions: Please address the study implications without overreaching what can be concluded from the data; the phrase "In this study, we observed ..." may be useful. Please interpret the study based on the results presented in the abstract, emphasizing what is new.

**Response:** We have addressed the study implications in the conclusions section (p3, lines 76–79).

5. Please conclude the Introduction with a clear description of the study question or hypothesis.

**Response:** We have concluded the Introduction section with study objectives (p5, lines 127 – 130).

6. For this observational study, in the manuscript text, please indicate: (1) the analytical methods by which you planned to test your hypothesis, (2) the analyses you actually performed, and (3) when reported analyses differ from those that were planned, transparent explanations for differences that affect the reliability of the study's results. If a reported analysis was performed based on an interesting but unanticipated pattern in the data, please be clear that the analysis was data-driven.

**Response**: We originally planned to identify predictors at baseline of HIV incidence using Cox proportional hazard models and Poisson regression. Based on one of peer-reviewers comment, highlighting the need for taking into account clustering by clinic, we have now analysed associations of HIV incidence with baseline factors using mixed-effects Weibull proportional hazard models, and time-updated factors using mixed-effects Poisson regression. Results are similar. Please see our response below (point 11). We have stated this in the manuscript.

7. Did your study have a prospective protocol or analysis plan? Please state this (either way) early in the Methods section.
a) If a prospective analysis plan (from your funding proposal, IRB or other ethics committee submission, study protocol, or other planning document written before analyzing the data) was used in designing the study, please include the relevant prospectively written document with your revised manuscript as a Supporting Information file to be published alongside your study, and cite it in the Methods section. A legend for this file should be included at the end of your manuscript.

**Response**: We have stated in the Methods section that the design and methods protocol of AURAH2 study had been previously published (line 133, reference no.10: Sewell et al, 2016 – *JMIR Research Protocols*). The study protocol doesn’t include an analysis plan for incidence analysis, therefore we have now included an analysis plan document with this revised manuscript submission (S1 Analyses Plan). This is now stated in the methods (p9, lines 283).

b) If no such document exists, please make sure that the Methods section transparently describes when analyses were planned, and when/why any data-driven changes to analyses took place.

**Response**: Please see our response above (point 7a).

c) In either case, changes in the analysis-- including those made in response to peer review comments-- should be identified as such in the Methods section of the paper, with rationale.

**Response:** We have described this in the methods section (p9, lines 282 – 286).

8. Please ensure that the study is reported according to the STROBE guideline, and include the completed STROBE checklist as Supporting Information. Please add the following statement, or similar, to the Methods: "This study is reported as per the Strengthening the Reporting of Observational Studies in Epidemiology (STROBE) guideline (S1 Checklist)."

**Response**: We have included STROBE checklist with this submission, and also added the requested statement to the methods section (p6, lines 150 – 152).

9. Your study is observational and therefore causality cannot be inferred. Please remove language that implies causality, such as “predictive” or “due to”. Refer to associations instead.

**Response:** We have now removed language that implies causality in our manuscript and refer to associations instead.

10. How was race/ethnicity defined and by whom? Why was race/ethnicity considered important in this study and what it is believed to represent [eg, are SES or genetic differences being attributed to race/ethnicity?]

**Response:** Race or ethnicity was self-reported by participants. Participants were asked at enrolment about how they define their ethnicity among these options: White (British, Irish, other); Black or Black British (Black African, Black Caribbean, other); Asian / Asian British (Indian, Pakistani, Bangladeshi, other); Mixed (White and Black African, White and Black Caribbean, White and Asian, Mixed other); Chinese and other.

In the UK in 2019, more than 50% new HIV diagnoses among GBMSM were among White ethnicity, the rest receiving care for HIV were from Black, Asian, and other minority ethnic groups.

11. In your statistical analyses, please use hierarchical/ multilevel models given that nationwide data is likely clustered at various county/ regional levels. The potential clustering of data (e.g., among patients from the same locality or hospital) would result in spurious effect estimates and standard errors.

**Response:** We have analysed associations of HIV incidence with baseline factors using mixed-effects Weibull proportional hazard models, and time-updated factors using mixed-effects Poisson regression. These take account of clustering according to clinic. Results are similar to those using Cox proportional hazard models and Poisson regression, therefore, our findings and conclusions are unchanged. We have also made changes to results (in tables and texts) and methods section – statistical analysis.

12. In statistical methods, please refer to any post-hoc corrections to correct for multiple comparisons during your statistical analyses. If these were not performed please justify the reasons.

**Response:** We did not correct p-values for multiple testing. Our analyses were pre-specified exploratory analyses and we did not consider it was necessary to make adjustment.

13. Please describe how you selected your adjustment variables.

**Response:** Adjustment variables were determined a priori and were socio-demographic characteristics that were considered not to be influenced by HIV incidence and sexual behaviour, but could have an impact on HIV incidence: time-updated age, country of birth and ethnicity, sexual identity, and university education. We have added this information to the manuscript (p8, lines 262 – 264).

14. Please specify the statistical test used to derive a p-value.

**Response:** For the hazard ratio of incidence, the likelihood-ratio test compares the random-effects model with a survival model with fixed-effects only.

15. Please define the abbreviations in tables and/or Figures e.g. PY, GBMSM, CLY, PHQ-9, GAD7

**Response:** We have now defined these abbreviations in tables and figures.

16. Please include p-values in table 2

**Response:** No results from hypothesis tests are reported in Table 2. We have added a p-value for trend in incidence in figure 1.

17. Please change p <0.0001 to P < 0.001.

**Response:** We have changed p<0.0001 to p<0.001.

18. Please provide titles and legends for all figures (including those in Supporting Information files).

**Response:** We already provided titles and legends for all figures, including supplementary materials.

19. Please update the access or retrieval date for all website citations to ensure that websites are still available

**Response:** all retrieval dates of access for all website citations have been updated. All websites are still available.

20. Please use the "Vancouver" style for reference formatting, and see our website for other reference guidelines

**Response:** We have changed our referencing formatting to the “Vancouver” style.

21. Please replace “injection drug users” with “people who inject drugs.”

**Response:** We have now replaced ‘injection drug users’ with ‘people who inject drugs’ in the manuscript.

**Comments from the reviewers:**

**Reviewer #1:**

This study reports trends in HIV incidence between 2013 and 2019 and the association of baseline and time-updated factors (demographic, socioeconomic, behavioural, lifestyle and health related) with HIV incidence, among a cohort of GBMSM in the AURAH2 prospective study.

**Comments:**

1. There are some typos in the text that need remedying.

**Response**: Thank you. We have made corrections to some typos in the manuscript (on 🡪 one, exclude 🡪 excluded).

2. "Supplementary material 1: Baseline socio-demographic, health and lifestyle characteristics, sexual behaviour, and PrEP and PEP use among participants who completed the baseline, four-monthly, and annual questionnaire in the AURAH2 study, 2013 - 2018"

Did the authors consider completing a comparison between groups, which may be insightful to gauge potential response bias?

It is noted that the groups in Supplementary material 1 are not independent groups, and so the authors may choose to separately amend the structure of the groups in order to draw comparisons (baseline only compared with baseline + four-monthly follow-up, for example).

**Response:** Thank you. We compared differences in socio-demographic characteristics and partnership status, among men who completed only baseline questionnaire (n=540) with those who continued on the study by completing at least an online questionnaire (n=622) in this study. Men who completed only the baseline questionnaire were significantly younger, had less financial security, had less stable housing status, less likely to have completed university level education, and less likely to be employed.

We have added to the S1 table, the p-values from chi-square test of differences between groups, and described this in the results section (p14, lines 320 – 323).

3. Overall, the authors have applied technically appropriate statistical methods, and presented the study outcomes in a clear and concise way. The study limitations have been suitably acknowledged in the discussion section.

**Response**: We thank reviewer #1 for the constructive and encouraging feedback.

**Reviewer #2:**

This is a timely analysis of changes in HIV incidence, sexual risk behavior, and PrEP use in a cohort of initially seronegative GBMSM from 3 urban STD clinics in the UK. The results are encouraging and demonstrate declining incidence as well as a decrease in some high-risk sexual behaviors, with concomitant increase in PrEP use. While there are limitations of the work, it represents an important contribution to our knowledge of changing HIV risk among GBMSM globally. I have a few queries and suggestions for the authors to consider, in order to improve clarity of the manuscript.

**Response**: We thank reviewer #2 for the detailed feedback and for emphasising that our study has an important contribution to the knowledge of changing HIV risk among GBMSM.

**Queries and suggestions:**

1. Methods, study design and participants. What is the justification for combining years in two of the categories (2013/2014 and 2018/2019). One can guess, but this should be explicitly stated.

**Response**: Thank you. We combined data for calendar year of 2013 and 2014 as one calendar period because recruitment of the AURAH2 study started on July 30, 2013, and so the calendar year of 2013 was less than six months; the number of men recruited by the end of 2013 (therefore the person-years) was too small to have a separate period. The same applied for calendar year of 2018 and 2019, we combined data because follow-up calculation was censored on June 30, 2019, therefore calendar year of 2019 was only six months.

We have now stated this in the methods section (p8, lines 256 – 258).

2. Methods, baseline measures. Were participants asked about sexual role taking, such as top/insertive, bottom/receptive, or versatile? If not, this should be mentioned as a limitation of the questionnaires used.

**Response:** Thank you. Yes, participants were asked about sexual role taking both at baseline and follow-up questionnaire: ‘In the past three months, when you had anal sex without a condom, which partner were you?’

We have added new sexual behaviour characteristic of ‘sexual role CLS in the past three months’ to the analyses and added results to the tables (Table 1, Table 3, S2 Table) and text (abstract lines 64 – 65; results lines 364 – 365 and 420). We have also revised our methods section – measures (p 6 – 7). We found significant associations between HIV incidence and versatile sexual condomless sex position, both in the baseline analysis and time-updated analysis.

3. Methods, p. 6 lines 155-157. The cutpoints for PHQ-9 and GAD-7 need a justification. This should be easy, as both are "moderate or severe" categories, but an explanation is needed for readers not familiar with these scales and their scoring. Similarly, the selected cutpoint for AUDIT-C needs justification and a reference.

**Response:** Thank you. We have now added a brief explanation about the scoring of PHQ-9, and GAD-7, and the justification of selected WHO AUDIT-C cut point (p7, lines 201 - 209).

4. Methods, p. 6, lines 158-160. The handling of missing data needs more justification. Why considering missing responses as "no" responses for behavior, mental health, and alcohol consumption and other variables as missing = excluded? Were data likely to be missing at random? Was imputation considered as an option to reduce bias due to missingness?

**Response**: Thank you. We did not exclude missing responses and replaced missing answers for sexual/HIV related behaviour, including PrEP and PEP use, with ‘no’ because our outcome of interests was ‘past report of sexual/HIV related behaviours’. Where there was no report of those measures in the questionnaire, including missing, we classified answers as the absence of the event. For instance, a missing response for CLS was considered to indicate that no CLS had been done in the past three months and a missing response for recreational drug use was considered to indicate that zero drugs had been taken. This was done as there appeared to be a common response pattern in which only those behaviours that had been done were ticked. Initial analyses that were undertaken to investigate whether excluding missing values (when defining each variable) had an impact on findings, demonstrated that this was not the case.

For socio-demographic factors such as age, ethnicity and country of birth, housing status, money status, etc, that could not be treated as ‘yes’ and ‘no’, we excluded missing responses from the analysis. The proportion of missing responses was low (<5% for each variable) so we elected not to perform imputation. We have now mentioned this in the text (p7, lines 211 – 214).

5. Results, lines 233-235. It is said that 622 of 1162 completed at least one online follow-up questionnaire, of whom only 483 completed at least one online follow-up questionnaire. How is this possible? Please edit for clarity. It looks like 622 were included in the analysis of time-updated variables.

**Response:** Thank you for the correction. It should be ‘483 men completed at least one **annual** follow-up questionnaire’ not **online**. We have replaced ‘online’ with ‘annual (p9, line 319).

6. Discussion, PrEP use. How might an approach combining PrEP and CLS to develop an "unprotected sex" variable change results. Did PrEP use match risk in this study or was it underutilized by some groups?

**Response:** Thank you. Yes, high-risk sexual behaviours (CLS, CLS with two or more partners, group sex, and using non-injection chemsex related drugs) were associated with PrEP use among men in this study, and PrEP use was also lower among those with lower socioeconomic status than among those of higher socioeconomic status (this has been reported previously, Hanum et al, 2020 – *The Lancet Public Health*).

The approach of combining PrEP and CLS to develop an ‘unprotected sex’ is a very good suggestion to consider, however, due to the difference in recall period of the two variables (PrEP use in the last 12 months and reported at the most recent annual questionnaire, while CLS in the past three months), we didn’t include in our analyses.

7. Discussion, injection drug use. The degree to which risk is increased with injection drug use in this cohort is noteworthy. Do GBMSM who inject drug have access to needle exchange and opioid substitution therapy in the UK? Is there any information on the extent to which men in this cohort accessed such programs?

**Response:** Thank you. Yes, GBMSM in the UK have access to harm reduction interventions such as needle and syringe programmes, opioid substitution treatment, and other treatments for drug misuse. Unfortunately, we didn’t collect data on the extent to which men in this cohort accessed such programs. We have added a brief sentence about this to the text (p20, line 487).

8. Discussion, limitations, lines 416-418. Please clarify the writing with respect to the 400 participants who are said to have completed "at least an online questionnaire" and were engaged in the study throughout. How does this relate to the earlier statements that 622 of 1162 completed at least one online follow-up questionnaire, of whom only 483 completed at least one online follow-up questionnaire.

**Response:** Thank you. Apologies for the confusion. We have clarified that the 483 men were those who completed at least an annual questionnaire. Please see our previous response (point 5) above. We have clarified this in the text.

9. Discussion, limitations. Could the authors be more specific about how the sociodemographics of the men participating in this cohort compare to those of new HIV cases among GBMSM in the UK? Which specific groups of men are underrepresented and warrant more targeted approaches? It would be nice to see some discussion on how this work relates to current known disparities in access to HIV services (for both positives and negatives) in the UK. Where might treatment as prevention and PrEP be falling short of their promise?

**Response:** Participants in AURAH2 at enrolment were representative of clinic attendees in England (compared to data from ‘Attendance of MSM at GUM services in England, 2018 – STI) based on median of age and ethnicity. Median age of MSM attending GUM services in England was 35 years (AURAH2 cohort 34 years), and 85% White (AURAH2 cohort 82%).

When comparing to the characteristics of MSM with HIV diagnosis in the broader UK that comprised of only around 50% of White ethnicity, it seems that ‘other’ ethnicity was under represented. We stated in limitations that our cohort that were predominantly of White ethnicity may not be representative of the broader population in the UK (p21, lines 518 – 520).

In the UK in general, although there has been steady progression in implementing combination prevention measures, and the efforts are having a major effect, there still remain opportunities for further improvements in increasing HIV test coverage among MSM. The majority of MSM in England appear to be engaged with sexual health services, however less than half of MSM have had HIV test in the last year. Our study re-emphasizes the importance of routine testing, including among minor ethnicity (p21, lines 546 – 548).

**Minor suggested corrections or edits:**

1. General. Consider whether condomless anal sex is best abbreviated "CLAS" or "CAS" rather than "CLS."

**Response:** Thank you for the suggestion. In the revised manuscript we prefer to keep using the ‘CLS’ term. We have mentioned and explained in the text that this term refers to ‘condomless anal sex’ only (p6, line 183).

2. Methods, line 159. "missing responses were considered to indicate that the absence…" Remove "that."

**Response:** Thank you. We have removed ‘that’ from the sentence (line 211).

3. Methods, line 189. "were include" should be "were included."

**Response:** Thank you. We have now replaced ‘include’ with ‘included’ (line 246).

4. Results, line 227. "taken at least on chemsex-related drugs" needs editing.

**Response:** Thank you. We have replaced ‘on’ with ‘one (line 297).

5. Table 1, country of birth and ethnicity. Suggest changing "Born on the UK, other ethnicity" to "Born in the UK, other ethnicity.

**Response:** Thank you. We have replaced ‘on’ with ‘in’ in Table 1.

6. Table 1 footnotes. Consider changing from "No group includes…" to "The no employment group includes…"

**Response:** Thank you. We have added ‘employment’ to the table 1 footnotes.

7. Supplementary Table 2. For consistency, CLS in the past 3 months should be bolded.

**Response:** Thank you. We have bolded the adjusted HR for CLS in the past three months in supplementary table 2.

8. Figure 2 caption. It is said in the text that annual questionnaire data were excluded for bacterial STI due to a difference in recall periods. Please correct the Figure 2 caption to reflect this.

**Response:** Thank you. For bacterial STIs, data were not included from the **baseline** questionnaire (since 2013), not **annual** questionnaire. We already stated this in the caption: **‘*bacterial STI in the previous 3 months, data from 4-monthly and annual questionnaires (N=3277 questionnaires)’***

**Reviewer #3: Linda Anne Selvey**

Thanks for this manuscript, which has some useful information that confirms the findings of others. I have a number of comments/suggestions below:

1. Methods:

More detail is required in the methods in relation to the analysis, particularly in terms of missing values. With missing values in the multivariable analysis, was the whole line (ie data about a single individual) excluded from the analysis, or were the missing values handled in a different way. This needs to be explicit in the methods. The total number of observations in the final model should also be included in the results section, not just in table 3.

**Response:** Thank you. In the multivariable analysis, the whole line for a single individual was excluded from the analysis if a value for one of the covariates is missing (complete case analysis). We have added a brief sentence about this to the method (p8, lines 279 – 281), and included the total number of observations to the text (p11, lines 413 – 414).

2. Also in the methods there is a description of the categorisation of the CLS and new sexual partners variables. How were these classifications made and why were these categories chosen. Zero to 10 new sexual partners in the last 12 months seems like a wide category. Why not have a variable with just Zero or one new partners?

**Response:** Thank you. The classifications of (number of partners of) CLS and new sexual partners had been pre-specified before and were asked in the questionnaires, with options of categories as shown in the tables. Number of new sexual partners refers to any new partners involved in sexual intercourse, both anal or vaginal, with or without condom; while number of CLS partners was specifically refers to number of partners of condomless anal sex. We are keeping these classifications as these have been pre-specified and categorised as the original questions.

3. Editing:

The paper needs a bit of an edit. For example line 189 (include vs included); line 227 (on vs one); line 236 (no full stop after 'study')

**Response:** Thank you. We have made corrections to these typos, please see our response to reviewer #2 above.

4. Discussion:

There were very small numbers of HIV infections in each calendar year, therefore with wide confidence intervals. This should be included as a limitation in the discussion.

**Response:** Thank you. We have included this in the limitation (p13, lines 520 - 523).
