# Supplementary material for: Trends in HIV incidence between 2013–2019 and association of baseline factors with subsequent incident HIV among gay, bisexual, and other men who have sex with men attending sexual health clinics in England: A prospective cohort study
Source: PLoS Med. 2021 Jun 18;18(6):e1003677. doi: 10.1371/journal.pmed.1003677 (PMC8253400; doi:10.1371/journal.pmed.1003677)
Supplement: Attachment — Submitted filename: Responses_AURAH2_HIV Incidence 2013-2019_PlosMed_R2.docx [file pmed.1003677.s007.docx]

**Manuscript PMEDICINE-D-21-01009R2**

**Response to the editorial team and reviewers**

**Requests from the editors:**

1. In the author summary, under the sub-heading, “what do these findings mean?”, please remove language that implies attribution or cause. Use phrases such as “may potentially be attributed to…” In the second bullet point replace the word “should” with “may potentially” or “could” or similar.

**Response:** Thank you. We have made corrections to the author summary sections as suggested by the editors (lines 94 – 97).

2. Please replace “injection drug users” with “people who inject drugs.” For example, line 272.

**Response:** Thank you. We have replaced ‘injection drug users’ with ‘people who inject drugs’ (line 276).

3. Please remove the “&” in reference # 11.

**Response:** Thank you. We have made correction to reference #11 (line 594).

**Comments from the reviewer:**

**Reviewer #2:**

The revision has addressed prior comments, and I believe the article is acceptable in its present form. However, I would also suggest that the authors consider reducing the numeric results in the abstract, dropping either the numerators and denominators, the p values, or both, to make the abstract findings more readable and potentially include some of the other interesting findings, such as the increase in PrEP use, decrease in PEP and some risk behaviors, but lack of association of PrEP with the outcome.

**Response**: We thank reviewer #2 for the positive feedback. We appreciate the reviewer’s comment to reduce the numeric results in the abstract, however, to include the actual amounts (%) or relevant outcomes and to quantify the main results with p-values in addition to 95% CI were compulsory requests from the editorial team. Therefore, we are keeping them in abstract. We also agree that including other findings will make the abstract more interesting, however, the abstract should not exceed 500 words. We could not add more results to the abstract.

I also note two very minor edits that are needed, below.

**Minor edits:**

1. Abstract: In the very long sentence on baseline associations, an extra "and" appears before "previous 12-month report of a bacterial STI diagnosis" and there is no HR with 95% CI provided for the finding on new sexual partners.

**Response**: Thank you. We have removed the extra ‘and’ and added the conditional hazard ratio for new sexual partners to the abstract (lines 58 – 61).

2. on p.14 under table 2, the sentence starting "90% men" needs an edit. Perhaps "The vast majority (27/30 or 90%) of men newly diagnosed with HIV were of White ethnicity."?

**Response**: Thank you. We have removed the sentence, as that sentence could be a misleading comment, given that 81% of the study population were of white ethnicity.
