# Supplementary material for: Trends in HIV incidence between 2013–2019 and association of baseline factors with subsequent incident HIV among gay, bisexual, and other men who have sex with men attending sexual health clinics in England: A prospective cohort study
Source: PLoS Med. 2021 Jun 18;18(6):e1003677. doi: 10.1371/journal.pmed.1003677 (PMC8253400; doi:10.1371/journal.pmed.1003677)
Supplement: Attachment — Submitted filename: Response_AURAH2Incidence_PlosMed_R3.docx [file pmed.1003677.s008.docx]

**Manuscript PMEDICINE-D-21-01009R3**

**Response to the editorial team**

**Final requests from the editors:**

1. Please remove the first mention of "prospective cohort" from the title. Kindly consider revising the title to, "Trends in HIV incidence between 2013 – 2019 and baseline predictors among Gay, Bisexual, and Other Men Who Have Sex with Men attending sexual health clinics in England: A prospective cohort study"

**Response:** Thank you. We have now removed ‘prospective cohort’ and ‘predictors’ from the title and we have revised the title as well (p1, lines 1 – 4; and p2, lines 27 – 29).

2. For consistency, please replace the term "predictors" with "factors" in the title and throughout the manuscript and tables.

**Response:** Thank you. We have now replaced the term ‘predictors’ with ‘factors’ in the title and throughout the manuscript and tables:

- abstract**:** line 32 and 34
- statistical analysis: line 223
- discussion: line 477, and 500.

We didn’t find any ‘predictor’ term in the tables.
